# Comparative Analysis of Different Univariate Forecasting Methods in Modelling and Predicting the Romanian Unemployment Rate for the Period 2021–2022

**DOI:** 10.3390/e23030325

**Published:** 2021-03-09

**Authors:** Adriana AnaMaria Davidescu, Simona-Andreea Apostu, Andreea Paul

**Affiliations:** 1 Department of Statistics and Econometrics, Bucharest University of Economic Studies, 010552 Bucharest, Romania; simona.apostu@csie.ase.ro; 2 National Scientific Research Institute for Labor and Social Protection, 010643 Bucharest, Romania; 3 National Institute of Economy, 050711 Bucharest, Romania; 4 Department of International Economic Relations, Bucharest University of Economic Studies, 15–17 Dorobanti St., Sector 1, 010404 Bucharest, Romania; andreea.paul@inaco.ro or; 5 President of Think-Tank INACO—The Initiative for Competitiveness, 030237 Bucharest, Romania

**Keywords:** unemployment rate, SARIMA, SETAR, Holt–Winters, ETS, neural network autoregression, Romania

## Abstract

Unemployment has risen as the economy has shrunk. The coronavirus crisis has affected many sectors in Romania, some companies diminishing or even ceasing their activity. Making forecasts of the unemployment rate has a fundamental impact and importance on future social policy strategies. The aim of the paper is to comparatively analyze the forecast performances of different univariate time series methods with the purpose of providing future predictions of unemployment rate. In order to do that, several forecasting models (seasonal model autoregressive integrated moving average (SARIMA), self-exciting threshold autoregressive (SETAR), Holt–Winters, ETS (error, trend, seasonal), and NNAR (neural network autoregression)) have been applied, and their forecast performances have been evaluated on both the in-sample data covering the period January 2000–December 2017 used for the model identification and estimation and the out-of-sample data covering the last three years, 2018–2020. The forecast of unemployment rate relies on the next two years, 2021–2022. Based on the in-sample forecast assessment of different methods, the forecast measures root mean squared error (RMSE), mean absolute error (MAE), and mean absolute percent error (MAPE) suggested that the multiplicative Holt–Winters model outperforms the other models. For the out-of-sample forecasting performance of models, RMSE and MAE values revealed that the NNAR model has better forecasting performance, while according to MAPE, the SARIMA model registers higher forecast accuracy. The empirical results of the Diebold–Mariano test at one forecast horizon for out-of-sample methods revealed differences in the forecasting performance between SARIMA and NNAR, of which the best model of modeling and forecasting unemployment rate was considered to be the NNAR model.

## 1. Introduction

Unemployment is a socio-economic problem facing all countries of the world, affecting both the standard of living of the people and the socio-economic status of the nations. Unemployment represents the result of a poor demand in the economy; a low demand implies a lower need for labor, which will lead either to reduced working hours or redundancies. Although unemployment is a consequence of a fundamental change in an economy, its frictional, structural, and cyclical behavior contributes to its existence.

The pandemic led to a large number of unemployed in Romania; in March, the unemployment rate rose to 4.6% compared to 3.9% in February 2020. The provisions of the military ordinances on stopping the spread of the new coronavirus have led many companies to partially or completely cease operations, which has led to the highest unemployment rate in the last two years.

Given the pandemic context, the unemployment rate in March was still low, due to the fact that in March, the effects of the pandemic were not entirely felt; companies waited until the last moment to see what measures the state would take to support technical unemployment. The first measures taken by companies were rest leave and other types of leave to be granted to employees.

The projection was that the unemployment rate in 2020 will increase, the month of March being only the beginning of the health crisis in Romania. According to the Ministry of Labor and Social Protection, more than 276,000 people were in a position where their employment contract was terminated on 30 April 2020. The industries with the most terminated employment contracts were wholesale and retail trade, manufacturing, and construction.

Although the effects of the coronavirus crisis have been seen in the economy since the measures taken in March 2020, forecasts indicated that the highest level of unemployment will reach 3.98% in the second quarter of 2020. Even the most pessimistic forecasts indicated that the unemployment rate in 2020 will not exceed 7%. The explanations for these low values compared to real figures were given by the fact that the persons returned to the country and the persons with terminated employment contracts are not included in the number of unemployed; at the end of March 111,340 terminated employment contracts had been registered and 250,000 people returned to Romania from abroad. Another explanation is the fact that the labor market was not growing at all during the crisis; therefore, people were not searching for a job, which is an essential condition to be declared unemployed.

The crisis caused by the coronavirus affected activities in many sectors and the number of unemployed increased, but this has not been reflected by the unemployment rate, as the real number of those unemployed was not included in the reporting base. Therefore, unemployment was lower, but this was not real, as the unemployed were not included in the statistics but rather in structural unemployment: the employment rate was reduced.

In this context, it becomes even more important to be able to provide future predictions of unemployment rate, and in order to do that, different univariate forecasting models (seasonal model autoregressive integrated moving average (SARIMA), self-exciting threshold autoregressive (SETAR), Holt–Winters, ETS (error, trend, seasonal), neural network autoregression (NNAR)) have been applied in order to identify the most appropriate model and to forecast the future values of unemployment rate. In order to do that, the period January 2000 to December 2020 has been used and divided into two sub-samples: the in-sample data or the training dataset covering the period January 2000–December 2017 used in the model identification and estimation and the test dataset or the out-of-sample data covering the last three years, 2018–2020. The forecast of unemployment rate relies on the next two years, 2021–2022.

Analyzing the patterns of unemployment rate, the research aims to respond to the following questions: Does the unemployment rate exhibits a non-stationary nonlinear pattern? Does the unemployment rate exhibit a seasonal pattern? Do the more sophisticated methods such as SETAR, NNAR, or SARIMA performs better than simple methods (HW or ETS)? What is the univariate forecasting method that performs best within in-sample data? What is the univariate forecasting method that has the best performance for the out-of-sample dataset? Which is the method that best captures the pandemic shock?

What is the combination of methods that could offer reliable future values for the Romanian unemployment rate?

Relying on these questions, the following three main hypotheses can be formulated:

**Hypotheses** **1** **(H1).** *The Romanian unemployment rate exhibits a non-stationary nonlinear and seasonal pattern over the period January 2000 to December 2017*.

**Hypotheses** **2** **(H2).** *NNAR and SARIMA models registered the best out-of-sample forecast performance from the all four methods applied*.

**Hypotheses** **3** **(H3).** *The combination of NNAR and SARIMA models offers the best approach in forecasting the unemployment rate for 2021–2022*.

The paper is organized as follows. The literature review presents an overview of the most important studies regarding this topic of forecasting unemployment rate, while Section 3 is dedicated to the presentation of five different forecasting models (SARIMA, NNAR, SETAR, Holt Winters, ETS). Section 4 incorporates information related to the data used in the analysis and the main empirical results of all five forecasting methods. The last part of this section ends with the comparison of models forecasting performance both for in sample and out-of-sample datasets. The final section of the paper presents the main conclusions about the relevance of this research.

## 2. Literature Review

The phenomenon of unemployment is the result of the dysfunctions of the economy, in the field of employment, being present both in the period of market economy transition and in the period of economic growth [1]. Unemployment is a very important labor market issue, being a mismatch between the labor demand and supply. This indicator has major social and economic implications, being one of the factors to be examined in macroeconomic growth and very important in comparing the country’s economic performance from a work perspective [2], affecting people’s living standard and the nation’s socio-economic status.

In this context, unemployment represents one of the biggest social problems of the world, being present in each country, the intensity of the phenomenon differing according to the economic development of a society. Population growth implies an increase regarding workforce, the jobs being insufficient in the short term [3]. The adjustment of the economic structure, the education system, and the establishment of the specialty does not satisfy the needs of economic restructuring; the professional skills of the rural labor force cannot satisfy the demand for jobs, aggravating the severity of unemployment. One of the solutions to this problem is the establishment of an early unemployment warning system, the forecast being absolutely necessary [4].

Forecasting the unemployment rate is very important for many economic decisions, especially setting relative policies by the government. The unemployment rate is correlated to the economic development of a society; therefore, different forecasting techniques are used for its forecast, from the simple OLS (ordinary least squares) method to the GARCH (generalized autoregressive conditional heteroskedasticity) models and neural networks. The econometric models are often related to stationary time series, seasonality, and trend analysis, and exponential smoothening to the simple OLS technique including ARIMA (autoregressive integrated moving average) models [5].

The ARMA and GARCH models were used by Chiros [6] to predict the unemployment rate in the UK; Parker and Rothman [7] modeled quarterly unemployment rates using the AR model (2), Power and Gasser [8] highlighted that the ARIMA (1,1,0) model has better forecasting performance for unemployment rates in Canada. Etuk et al. [9] indicated that the ARIMA (1,2,1) model is suitable for forecasting the unemployment rate in Nigeria.

Rothman [10] used six nonlinear models for out-of-sample forecasting, Koop and Potter [11] used the autoregressive threshold (ART) for modeling and forecasting the monthly unemployment rate, and Proietti [12] used seven forecasting models (linear and nonlinear). Johnes [13] used autoregressive models, GARCH, SETAR (Self-Exciting Threshold AutoRegressive) and neural networks in order to predict the monthly unemployment rate in the United Kingdom, the SETAR model registering the best results. Peel and Speight [14] also concluded that the SETAR model is better, in terms of root mean squared error (RMSE), compared to AR models.

As an alternative to ARMA models, Gil-Alana [15] used an exponential Bloomfield spectral model to model unemployment in the UK, the results indicating that this model is suitable for forecasting this phenomenon.

Forecasting the unemployment rate in Italy, Naccarato et al. [16] used both official data and the Google Trends query rate, estimating two different models: ARIMA and VAR (vector-autoregressive models), the VAR model registering a lower forecast error.

The autoregressive integrated moving average (ARIMA) models were introduced by Box and Jenkins [17], also developing the practical process to select the most suitable ARIMA model. ARIMA models are more secure in case of short-term forecasts compared to long-term forecasts [18]. For seasonal and non-seasonal data, the SARIMA (seasonal model autoregressive integrated moving average) is used. The SARIMA model is an extension of the simple ARIMA models, being used for inflation forecasting [19,20,21], for exchange rate forecasting [22,23], for tourist arrivals and income forecasting [24,25], as well as for unemployment forecasting. The literature includes a lot of studies on forecasting using ARIMA models, respectively the Box–Jenkins methodology, which is widely used by many researchers to highlight future unemployment rates [26].

Among them, Wong et al. [27] developed autoregressive integrated moving average (ARIMA) models in order to analyze and forecast important indicators in the Hong Kong construction labor market: employment level, productivity, unemployment rate, underemployment rate, and real wage. Ashenfelter and Card [28] analyzed unemployment, nominal wages, consumer prices, and the nominal interest rate, using the autoregressive moving average model. Kurita [29] forecasted the unemployment rate using autoregressively integrated fractional moving average, the model being much better than naive predictions.

Predictions of unemployment rate in the world using the ARIMA model were made by Chih-Chou and Chao-Ton [30], Etuk et al. [22] and Nkwatoh [31] in Nigeria using the ARIMA and ARCH model, Kanlapat et al. [32] in Thailand, Nlandu et al. [33] in Barbados, using the seasonal integrated autoregressive moving average model (SARIMA), Dritsakis and Klazoglou [34] in the USA using SARIMA and GARCH models, and Didiharyono and Syukri [35] in South Sulawesi using the ARIMA model.

In the European Union, the unemployment rate is forecasted using Box–Jenkins and TRAMO/SEATS methods [36,37]. In European countries, the unemployment rate was predicted using the Box–Jenkins methodology in Germany using the ARIMA and VAR models [38], in the Czech Republic using the SARIMA model [39,40], in the German regions using a model spatial GVAR [41], in Greece, both as a dynamic process and as a static process using SARIMA models [42,43], and in Slovakia using ARIMA and GARCH models [44].

Unemployment predictions using VAR were realized also by Kishor and Koenig [45], taking into account that data are subject to revisions. The accuracy of forecasts based on VAR models can be measured using the trace of the mean-squared forecasts error matrix, generalized forecasts error second moment [46], transfer functions [47], and combined forecasts based on VAR models are a good strategy for improving predictions’ accuracy [48].

Wang et al. [49] used back propagation neural networks (BPNN) and the Elman neural network to predict unemployment rate. Neural networks are also used by Peláez [50] to forecast the unemployment rate, together with econometric models.

As the asymmetric behavior of unemployment rate can be modeled using a nonlinear time series model, Skalin and Terasvirta [51] proposed STAR. Peel and Speight [14] forecasted the unemployment rate in the UK using self-exciting threshold autoregressive (SETAR) models and an autoregressive model, in terms of RMSE, SETAR models registering better forecasting performance. Koop and Potter [11] used threshold autoregressive (TAR) in order to forecast the US unemployment rate, Johns [13] forecasted the unemployment rate using AR(4), AR(4)-GARCH(1,1), SETAR(3,4,4), and neural network, highlighting that SETAR is the best model.

According to the international definition [52], the unemployed are people aged between 15 and 74 who at the same time satisfy three conditions: they do not have a job, are available to start work in the next two weeks, and have been actively looking for a job anytime in the last four weeks. The unemployment rate represents the share of the unemployed in the active population, the active population in a country including all persons who provide labor available for the production of goods and services during the reference period, including employees and the unemployed.

Unemployment was first introduced in Romania in 1991, and the first study to assess unemployment according to ILO standards was conducted in 1994 [1]. Specific to a country in transition, unemployment in Romania was the result of the enterprise restructuring and the contraction of production [53].

In the first period after 1990, although many occupations appeared in Romania, the number of unemployed increased; 1994 had the highest registered unemployment rate [54]. In the period 1995–1996, the number of unemployed decreased by 46.28% and then increased significantly until 1999 due to socio-economic imbalances that arose from the closure of other productive structures. After 1999, the economic activities were restructured and privatized, especially in the case of large companies, leading to large layoffs, but also to the emergence of new jobs, the result being the unemployment reduction. Since 2000, employment in Romania has registered a continuous increase, with small fluctuations, leading to a reduction in unemployment [55].

In order to substantiate the macroeconomic policies in Romania, it is important and topical to forecast the labor supply, employment, and unemployment. In Romania, as in other European countries, unemployment is monitored and assessed very seriously. The most common method used in order to predict the unemployment in Romania involves ARIMA models.

Son et al. [56] analyzed the unemployment rate in EU-27 countries, focusing on Romania, concluding that the unemployment rate can be modeled by using a linear autoregressive model. Others studies using ARIMA models in order to predict the unemployment rate in Romania were realized by Madaras [57], Bratu [58], and Simionescu [59], while Dobre and Alexandru used the VARMA and VAR models [60], and at the level of two Romanian counties (Brasov and Harghita), studies used the Box–Jenkins methodology and NAR model based on the artificial neural network. Comparing the forecasted values with the officially recorded unemployment rate from the same period, we noticed that by the end of the period, the differences between the real and the predicted values became larger in the NAR model than in the ARMA model forecast, medium-term forecasts, forecasts based on the ARMA model being more accurate.

Other forecasts of the unemployment rate in Romania were realized by Bratu and Marin [61] using several techniques: econometric, exponential modeling, smoothing technique, and moving average method; of these, predictions based on the exponential smoothing technique recording the highest degree of accuracy. Voinegu et al. [62] predicted the unemployment rate using Holt’s improved model, the monthly series being constructed and disseminated in three forms: adjusted, seasonally adjusted, and trend adjusted. Other predictions used the Kalman approach, the Kalman filter being appropriate for calculating the natural unemployment rate [63]. In the short term, Zamfir [64] modeled the unemployment rate using stochastic models.

Simionescu [65] predicted the unemployment rate in Romanian counties using Internet data and official data as well as a methodology consisting of different types of models with panel data. In the case of the quarterly unemployment rate, updated vector-autoregressive models (VAR models) and a Bayesian VAR model were used, but the VAR model exceeded the Bayesian approach in terms of predicted accuracy [66].

In order to analyze the dynamics of the unemployment rate in Eastern Europe, including Romania, Lukianenko et al. [67] constructed econometric regression models with nonlinearities due to discrete changes in modes. Using the Markov switching model, regularities were captured by modeling the asymmetry in the unemployment rate during contractionary states, revealing the specifics of the labor market for each country and the differences in the flexibility of reactions to changes in the economic environment.

## 3. Data and Methodology

In order to determine the best model to forecast the Romanian unemployment rate, we have investigated the monthly unemployment rate covering the period 2000M01 to 2020M12. The data were provided by Eurostat (European Union labour force survey, EU-LFS).

When choosing models, it is common practice to split the available data into two portions, training and test data, where the training data are used to estimate any parameters of a forecasting method and the test data are used to evaluate its accuracy. Therefore, the training set or “in-sample data” was set to the period 2000M01–M2017M12, and the test set or the “out-of-sample data” was set to the period 2018M01-2020M12. The forecast of unemployment rate will rely on the next two years of the period 2021–2022.

The main objective of the paper is to compare the forecasting potential of five models: exponential smoothing models (additive and multiplicative Holt–Winters (HW) models, and ETS model), the SARIMA model, the neural network autoregression (NNAR) model, and the SETAR model, and to predict future values of unemployment rate beyond the period under consideration.

Therefore, with the study, the forecasting performance was derived from the five models in view of identifying the best suited forecasting procedure for the monthly unemployment rate, taking into account the following steps:

1. Fit the Holt–Winters models (additive and multiplicative) on the training dataset (January 2000 to December 2017)
2. Fit the ETS model on the training dataset
3. Fit the NNAR model on the training dataset
4. Fit the SARIMA model on the training dataset
5. Fit the SETAR model on the training dataset
6. Compare the in-sample forecast accuracy measures for the all models
7. Compare the out-of-sample forecast accuracy measures for the models over the period January 2018 to December 2020
8. Compare the forecast projections of unemployment rate for all models over the period January 2021 to December 2022.

### 3.1. Holt–Winters Method and ETS Models

We will start our technical demarche by introducing the class of exponential smoothing methods as widely used forecasting procedures referring particularly to the Holt–Winters (HW) method, which is a commonly used forecasting method in time series analysis incorporating both trend and seasonal components, irrespective of whether they are additive or multiplicative in nature. The additive method is preferred when the seasonal variations are roughly constant through the series, while the multiplicative method is preferred when the seasonal variations are changing proportional to the level of the series.

The Holt–Winters’ additive method can be written as follows:

(1) $$L_{t}=\alpha \left(y_{t}-S_{t-s}\right)+\left(1-\alpha \right)\left(L_{t-1}+b_{t-1}\right)$$

(2) $$b_{t}=\gamma \left(L_{t}-L_{t-1}\right)+\left(1-\gamma \right)b_{t-1}$$

(3) $$S_{t}=\delta \left(y_{t}-L_{t}\right)+\left(1-\delta \right)S_{t-1}.$$

The Holt–Winters’ multiplicative method can be written as follows:

(4) $$L_{t}=\alpha \frac{y_{t}}{S_{t-s}}+\left(1-\alpha \right)\left(L_{t-1}+b_{t-1}\right)$$

(5) $$b_{1}=\gamma \left(L_{t}-L_{t-1}\right)+\left(1-\beta \right)b_{t-1}$$

(6) $$S_{t}=\delta \frac{y_{t}}{L_{t}}+\left(1-\delta \right)S_{t-1}$$

where *t* = 1, …, *n*, *s* represents the length of seasonality (months), *L_t_* represents the level of the series, and *b_t_* denotes the trend and *S_t_* seasonal component [22]. The constants used for this model are *α* (level smoothing constant), *γ* (trend smoothing constant), and *δ* (seasonal smoothing constant). In order to choose the most adequate smoothing constants, we tested different values of the smoothing constants. The model is selected according to the certain forecast accuracy such as MAPE (the mean absolute percentage error), the best model being the model who register the minimum value for MAPE.

The ETS (error, trend, seasonal) model represents time series models that support the exponential smoothing methods, consisting of a trend component (T), a seasonal component (S), and an error term (E). These are based on error–trend–season probabilities of Hyndman, being defined an extended class of ES methods using probability calculations based on the state space, with support for model selection and the calculation of standard forecast errors [68].

The long-term movement is characterized by the trend term, the pattern with known periodicity is reflected by the seasonal term, and the error term represents the irregular, unpredictable component of the series.

ETS models generate both point forecasts and prediction intervals (or forecast). If the same values of the smoothing parameters are used, the point forecasts are identical but will generate different prediction intervals.

The individual components of an ETS specification may be specified as being of the following form: N = none, A = additive, M = multiplicative:

E: A, M

T: N, A, M

S: N, A, M.

An ETS (A,A,A) decomposition is a Holt–Winters method with an additive seasonal component, and an ETS (M,A,M) represents a Holt–Winters method with a multiplicative seasonal component.

The automatic selection of the model is based on the ETS smoothing. For each ETS model, the probability and the forecast error can be calculated by comparing the information criterion based on probability or an out-of-sample AMSE (The average mean square error estimator finds the parameter values and initial state values that minimize the average mean square error of the step forecasts of the specified ETS model) in order to determine the model that best fits the most accurate data or forecasts. Automatic selection for unemployment rate forecasting using the ETS framework will be done using Akaike Information Criterion corrected (AICc) minimization.

### 3.2. The Neural Network Autoregression Model

**Figure 1:**
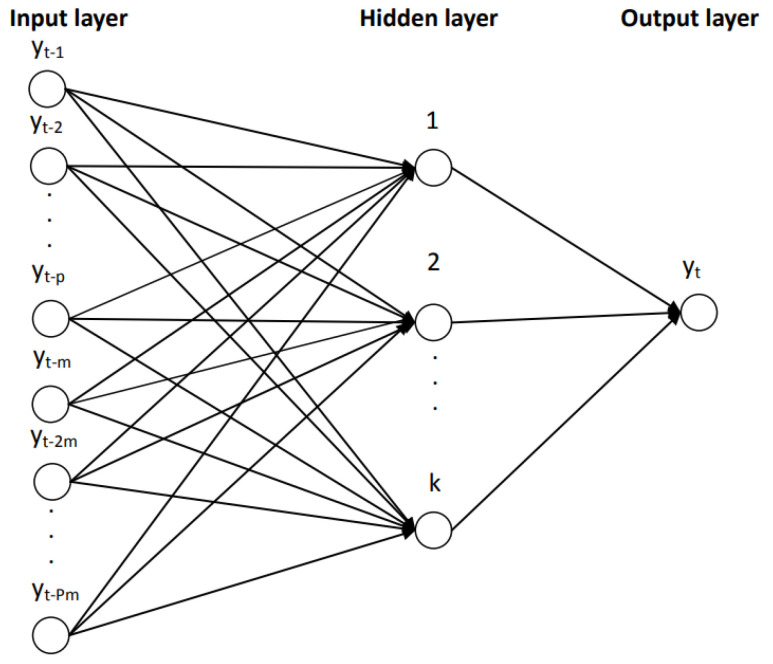
A diagrammatic representation of the NNAR(p,P,k)m model. Source: Touplan [69]. NNAR: neural network autoregression.

Artificial neural networks are used to model complex nonlinear relationships between input variables and output variables. An autoregression model of the neural network (NNAR) has delayed values of a time series as input in the model, and it predicted values of the time series as output. The major difference of the NNAR method compared to the HW method is the non-existence of the restriction of stationary parameters. Considering the seasonality of the monthly unemployment rate, the specification of the neural network will be NNAR(p,P,k)m, and the graphical representation from Figure 1. By adding an intermediate layer with hidden neurons, the neural network becomes nonlinear, and without the hidden layer, NNA(p,P,0)m becomes SARIMA(p,0,0) (P,0,0)m.

The NNAR model represents a feedforward neural network, involving a linear combination function and an activation function. The linear combination function has the following form [70,71]:

(7) $$net_{j}=\sum_{i}w_{ij}y_{ij}.$$

The hidden layer has a nonlinear sigmoid function in order to issue the input for the next layer:

(8) $$s\left(z\right)=\frac{1}{1+e^{-z}}.$$

In the case of NNAR(p,k) with p delayed entries and k nodes in the hidden layer, the model involves delayed time series values as entries in a neural network, considering a feed-forward network with a single hidden layer. The seasonal component is present in the data (m = 12), so the last observed values from the same season will be added as inputs, NNAR becoming NNAR(p,P,k)12.

The forecasting procedure is iterative; the one-step ahead forecast uses historical inputs; and the two-steps ahead forecast uses the one-step ahead forecast and the historical data.

### 3.3. Seasonal Autoregressive Integrated Moving Average Model (SARIMA) Model

Taking into account the seasonal pattern exhibited by the monthly unemployment rate, a seasonal process may be considered; therefore, the ARIMA model will become a SARIMA model. The seasonal autoregressive integrated moving average (SARIMA) model is a generalized form of an ARIMA model that accounts for both seasonal and non-seasonal data. The SARIMA model is denoted as ARIMA(p,d,q) (P,D,Q)S and has the following specification based on the backshift operator [72,73]:

(9) $$\phi \left(B\right)\phi \left(B^{s}\right){\left(1-B\right)}^{d}{\left(1-B^{s}\right)}^{D}Y_{t}=ϴ\left(B\right)ϴ\left(B^{s}\right)\varepsilon_{t}$$

(10) $$\phi \left(B\right)=1-\phi_{1}B-\phi_{2}B^{2}-\ldots -\phi_{p}B^{p}$$

(11) $$\phi \left(B^{s}\right)=1-\phi_{1}B^{s}-\phi_{2}B^{2s}-\ldots -\phi_{p}B^{2Ps}$$

(12) $$ϴ\left(B\right)=1+{ϴ}_{1}B+{ϴ}_{2}B^{2}+\ldots +{ϴ}_{q}B^{q}$$

(13) $$ϴ\left(B^{s}\right)=1+{ϴ}_{1}B^{s}+{ϴ}_{2}B^{2s}+\ldots +{ϴ}_{Q}B^{Qs}$$

where *Y_t_* represents the time series data at period t, *B* denotes the backshift operator, *ε_t_* is a sequence of i.i.d. variables (mean zero and variance σ^2^), *s* is the seasonal order, *ϕ_i_* and *ϕ_j_* are the non-seasonal and seasonal AR parameters, *ϴi* and *ϴj* are respectively non-seasonal and seasonal MA parameters, p, d, and q denote the non-seasonal AR, I, and MA orders, respectively, and P, D, and Q respectively represent the seasonal AR, I, and MA orders.

Similar to the Box–Jenkins methodology, also, the SARIMA model follows a five-step iterative procedure: identification, estimation, selection, diagnostics, and forecasting [34,60,69].

Before fitting a particular model to time series data, the stationarity of a series must be checked [74]. In order to identify if the time series in stationary, the graphical representation of the series together with the correlogram of the series in level, Bartlett test, and Ljung–Box test can be applied. In order to test if the series has a unit root, the Augmented Dickey–Fuller and Philips–Perron tests can be used. To obtain a stationary time series, the corresponding value of d is estimated, in the case of a non-stationary series in mean, the series is differentiated, and in the case of a non-stationary series in variance, the series is logarithmized.

In addition, the series needs to be tested against the presence of a structural break using the Zivot–Andrews test. The Zivot and Andrews endogenous structural break test is a sequential test that uses the full sample and a different dummy variable for each possible break date. The break date is selected where the t-statistics of a unit root ADF (Augmented Dickey Fuller) test is at a minimum (most negative). Consequently, a break date will be chosen when the null hypothesis of a unit root will be rejected. The Zivot–Andrews test uses three scenarios: a structural break in the level of the series, a one-time change in the slope of the trend, and a structural break in the level and slope of the trend function of the series. Therefore, under the test, the null hypothesis assumes that the series yt contains a unit root without any structural break, against the alternative that the series is a trend-stationary process with a one-time break occurring at an unknown time point.

Another important feature that needs to be investigated for a series exhibiting a seasonal pattern under the stationarity condition is to test for the presence of a seasonal unit root using the HEGY test [75]. The HEGY test is used in case of a seasonal and non-seasonal unit root in a time series. A time series *y_t_* is considered as an integrated seasonal process if it has a seasonal unit root as well as a peak at any seasonal frequency in its spectrum other than the zero frequency.

The test distinguishes between deterministic seasonality—which can be removed by seasonal adjustment—and stochastic seasonality—which refers to unit roots at the seasonal frequencies [76].

Once the stationarity has been achieved, the *identification stage* involves determining the proper values of p, P, and q, Q based on the correlogram of the stationary series (ACF and PACF plot). Checking the ACF and PACF plots, we should both look at the seasonal and nonseasonal lags. Usually, the ACF and the PACF have spikes at lag k and cut off after lag k at the non-seasonal level. The ACF and the PACF also have spikes at lag ks and cut off after lag ks at the seasonal level. The number of significant spikes suggests the order of the model [74].

An SAR signature usually occurs when the autocorrelation at the seasonal period is positive, whereas an SMA signature usually occurs when the seasonal autocorrelation is negative.

In the model selection stage, we need to decide on the optimal model from several alternative estimated models in the situation in which two or more models compete in the selection of the best model for the study.

In order to be able to make a decision, we can rely on the penalty information criteria (Akaike Information Criterion (AIC), the Akaike Information Criterion corrected (The AICc includes a penalty that discourages overfitting, and increasing the number of parameters improves the goodness of fit [72]) (AICc), and the Bayesian Information Criterion (BIC), choosing as an optimal model the model with the smallest values of AIC, AICc, and BIC.

In the model estimation stage, the parameters of the chosen model are estimated using the method of maximum likelihood estimation (MLE).

The diagnostic checking stage is the next stage investigating if the estimated model or models are firstly validated in accordance with the classical tests: t-test for the statistical significance of the parameters and F-test for the statistical validity of the model.

Secondly, the main hypotheses on the model residuals need to be tested, showing that they are white noise, homoscedastic, and do not exhibit autocorrelation. The normality of the residuals has been checked using Jarque–Bera test, while for non-autocorrelation, the Ljung–Box test has been applied. When the variance of the residuals is not constant, the issue of conditional heteroscedasticity is one of the key problems that is likely to encounter when fitting models. For checking autoregressive conditional heteroskedasticity (ARCH) in the residuals, the squared residuals correlograms and the ARCH-LM test can be used. In case there is no ARCH in the residuals, the autocorrelations and partial autocorrelations should be zero; regardless, the lags and the Q-statistics should be insignificant.

If at the level of this stage, one of the hypotheses is invalidated, we need to return to the first stage of the model and rebuild a better model. Otherwise, if the model passes this stage, the forecasting process can be implemented to predict future time series based on the most reliable model validated in the previous stages.

The final stage is forecasting in order to design future time series values, using the most convenient model according to previous stages [43].

### 3.4. SETAR Model

The SETAR model is part of the more general class of threshold autoregressive models (TAR) and represents an extension of autoregressive models, bringing as its main advantage in modeling a time series and a higher flexibility in parameters due to a regime-switching behavior. Thus, this particular type of model allows for the prediction of future values of unemployment rate, assuming that the behavior of the time series changes when the series switch the regime, and this switching is dependent on the past values of the series. The model relies on an autoregressive model of lags p, on each regime, and it is denoted to be SETAR(k,p), where k is the number of thresholds (k + 1 regime assumed in the model) and p is the order of an AR(p).

Even if the process is assumed to be linear in each regime, the switching from one regime to another transforms the process into a nonlinear one.

The general specification of a two-regime SETAR(2,p,d) of the following regime to the others proves the entire regime as nonlinear [66,67,73]. The two-regime version of the SETAR model of order p is given by:

(14) $$y_{t}=\phi_{0}^{\left(1\right)}+\sum_{i=1}^{p\left(1\right)}\phi_{i}^{\left(1\right)}y_{t-i}+\varepsilon_{t}^{\left(1\right)},\;\mathrm{if}\;y_{t-d\;}\leq \;\tau$$

(15) $$y_{t}=\phi_{0}^{\left(2\right)}+\sum_{i=1}^{p\left(2\right)}\phi_{i}^{\left(2\right)}y_{t-i}+\varepsilon_{t}^{\left(2\right)},\;\mathrm{if}\;y_{t-d\;}>\;\tau$$

where $\phi_{i}^{\left(1\right)}$ and $\phi_{i}^{\left(2\right)}$ are the coefficient in the lower and higher regime, respectively, which needs to be estimated; τ is the threshold value; p(1) and p(2) are the order of the linear AR model in the low and high regime, respectively. y_t-d_ is the threshold variable governing the transition between the two regimes, d being the delay parameter, which is a positive integer (d < p); $\varepsilon_{t}^{\left(1\right)}$ and $\varepsilon_{t}^{\left(2\right)}$ are a sequence of independently and identically distributed random variables with zero mran and constant variance [77].

The main phases for setting a SETAR model are the order selection of the model based on AR(p) order identification together with the test for threshold nonlinearity, model identification requiring the selection of the delay parameter d together with the location of the threshold value, model estimation and evaluation, and the last stage forecasting the future values of unemployment rate.

Thus, the first stage in applying the SETAR model is to analyze the existence of a nonlinearity behavior, and for that, it is important to first determine the appropriate lag length of the autoregressive model AR(p) for the analyzed time series, and the choice could rely on the minimum value of AIC. Secondly, we will test the existence of nonlinearity using the Tsay F test, the null hypothesis of linearity being rejected if the p-value of the test is smaller than the significance level assumed.

Proving that there is nonlinearity in the time series, we can pass to the second stage—model identification—and we will consider a two-regime SETAR model with the order p of autoregressive parts equal in both regimes, SETAR(2,p,d).

In the third stage, the selection of delay parameter together with the location of the threshold value is realized, taking into account that the possible value d is less than order. Therefore, several SETAR models with different delay parameters and threshold values can be identified, and based on a grid search method, the best model is selected to be the model with the smallest value for the residual sum of squares.

The model is estimated using the MLE, and then, the adequacy of the selected model is evaluated based on diagnostics tests on residuals. The ARCH-LM test is used for testing the hypothesis of constant variance and Breusch–Godfrey is used for testing for higher-order serial correlation in the residuals.

### 3.5. Forecasting Performance Comparison

In order to provide predictions of the future values of unemployment rate based on past and present data and analysis of trends, it is important to use both in-sample and out-of-sample forecasting performance methods, even if the out-of-sample is known to offer more reliable results. Therefore, a model with good performance in the out-of-sample forecasting performance is picked as the best model. The forecasting performance of models was evaluated on two sub-samples: in-sample data, 2000M01–2017M12, which is used to estimate and identify the model and also to provide in-sample forecasting performance, and out-of-sample data, 2018M01–2020M12, which is used for analyzing the forecasting performance.

Forecasting accuracy offers valuable information about the goodness fit of the forecasting model and shows the capacity of the model to predict future values of unemployment rate. Three criteria have been used to evaluate the performance of models both on in-sample data and out-of-sample data: the root mean squared error (RMSE), the mean absolute error (MAE), and the mean absolute percent error (MAPE). The better forecast performance of the model is that with the smaller error statistics.

Another test used to check the existence of differences between the forecast accuracy of two models was the Diebold–Mariano test [78], which assumes in the null hypothesis the absence of such a difference against the alternative of the existence of a statistical difference between the forecast accuracy of the models.

## 4. Data and Empirical Results

We have used in the empirical analysis the ILO unemployment rate for Romania covering the period 2000M01–2020M12, summing up a total of 252 monthly observations. The data source is the Employment and Unemployment database of Eurostat. We used for the model estimation and identification the estimation period 2000M1–2017M12 as training data and the period 2018M01–2020M12 as test data, while the forecast projections have been made for the next two years, 2021–2023.

The evolution of unemployment rate revealed an oscillating trend, from peaks (8.1% in January 2001 and January–March 2002) to minimum levels (5% in September 2008). The winter months of the years 2000, 2001, and 2002 registered increases in the unemployment due to lack of jobs, the year 2002 recording the highest rate of the monthly unemployment rate (144%). A potential explanation could be the dismissals that took place as a result of the implementation of restructuring and privatization programs of different sectors of activity. The impasse in the general economic and social development of Romania, the low living standard, and the lack of future perspectives from the period 1998–2000 reactivated the migration phenomenon, causing many Romanians to look for a job in more developed countries. However, after 1998, illegal migration predominated, which was mainly directed to Italy and Spain.

Compared to previous years, in 2004, the unemployment rate decreased; the number of persons entering unemployment was lower than the previous year by 92,442 persons. The 278,080 unemployed related to 2004 came from the redundancies that took place as a result of restructuring and privatization programs of different sectors of activity, and of these, only 67,042 people came from collective redundancies; the remaining 211,038 people came from current redundancies personal.

Young people represent the best professionally trained age group in Romania, but also the most exposed to unemployment, highlighting the brain-drain phenomenon. The decrease in the unemployment rate in the period 2002–2006 is due both to legal and illegal departures of persons to work abroad. Thus, in 2006, according to the figures offered by Eurostat, it was estimated that over two million Romanians work in the countries of Western Europe or other developed countries. The economic crisis from 2008 created another peak in the evolution of unemployment rate, registering in the first three months of 2010 the values of 7.7%, 7.7%, and 7.9% and oscillating around this value until the first three months of 2015 (7.5%, 7.4%, and 7.2%).

The unemployment rate in 2008 decreased compared to the previous year (6.4%), but during the economic crisis of 2008–2009, there was a substantial increase in the unemployment rate. Although the number of jobs in the economy is constantly decreasing, the unemployment rate is decreasing, the explanation of this paradox being given by the following:

1. Working abroad: according to official estimates, in the first nine months of 2010, the number of those who went to work abroad exceeded 380,000, of which 140,000 went on their own, 140,000 went through recruitment agencies, and 102,000 went through the NAE (National Agency for Employment)
2. Retirement of some of the employees. Quarterly, 70,000–80,000 people retire; therefore 200–300,000 employees must be replaced annually. It is very likely that companies will no longer replace some of the people who have retired, so that the number of employees can decrease without increasing the number of unemployed.
3. Undeclared work. In second quarter of 2010, the number of undeclared workers increased by almost 100,000.

For the last years, the trend for unemployment rate was continuously downward, with a minimum point in the first month of 2020 (3.8%), and since February 2020, the unemployment rate registered an ascendant trend. The reversed trend was due to the high unemployment rate (18.5%) among young people (15–24 years) and seasonality in the construction and tourism sectors.

In 2019, the unemployment rate decreased to 3.9%, compared to 4.2% in 2018, affecting to a greater extent the graduates of lower and secondary education, for which the rate was 6.3% and 4%, respectively, according to data from the National Institute of Statistics (NIS). On the other hand, the unemployment rate for people with higher education was much lower, 1.6% in 2019.

In 2020, in the context of the coronavirus crisis, the unemployment rate started to increase since February, with the taking of safety measures, reaching 5.2% in May, which was the highest level since 2017. According to the NIS, the number of unemployed people exceeded 460,000, with over 110,000 more people than the same period last year.

In August, the unemployment rate decreased by 0.1 points compared to the previous month, but it increased by 1.5 points compared to the same month last year. Thus, August was the first month since the beginning of the COVID-19 pandemic on the Romanian territory when the unemployment rate registered a decrease. In March, the unemployment rate was 4.6%.

In autumn, in October 2020, the unemployment rate increased by 0.2 points compared to the previous month (5.1%), unemployment among men being higher than among women by 0.5 percentage points, according to the NIS. Unfortunately, youth unemployment (18–24 years) is approaching 20%. As for the number of unemployed, Romanians looking for a job were 477,000, with over 100,000 more than in October of the previous year.

In January–October 2020, the medium unemployment rate stood at 4.9%, which was up 1.1 points year/year, an evolution determined by the incidence of the health crisis (and the consequences of this unprecedented shock), partially offset by the implementation of an unprecedented relaxed mix of economic policies.

**Figure 2:**
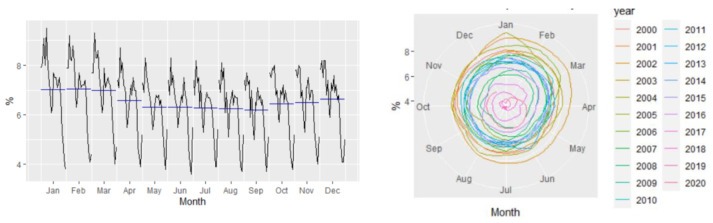
The seasonal pattern in the monthly ILO unemployment rate.

**Figure 3:**
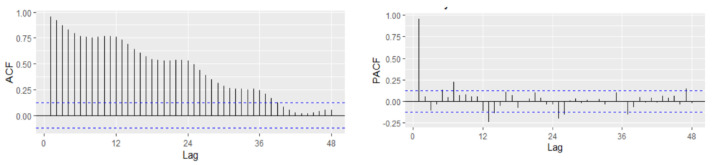
Autocorrelation and partial correlation plot of Romania’s monthly unemployment rate for the horizon 2000–2020.

Figure 2 revealed that the Romanian unemployment rate exhibited seasonal fluctuations over the period 2000–2020, with peaks in the last and the first months of the year. Figure 2 depicts the evolution of the monthly unemployment rate, revealing a clear seasonal component in the data, which was confirmed also by the autocorrelation plot (Figure 3).

### 4.1. Holt–Winters Results

The empirical results of Holt–Winters additive and multiplicative models revealed that because both models have exactly the same number of parameters to estimate, the training RMSE from both models can be compared, revealing that the method with multiplicative seasonality fits the data best. In addition, based on the informational criteria (AIC, AICc, or BIC), the optimal model is also the multiplicative version of HW. Table 1 gives the results of the both in-sample and out-of-sample forecasting accuracy measures of the Holt–Winters methods for the unemployment rate.

According to the RMSE measure, the multiplicative model performs better than the additive one, while based on the other forecast accuracy measures (MAPE, MASE, or MAE), the optimal model is the additive one, for which they registered the minimum values (Table 2).

**Figure 4:**
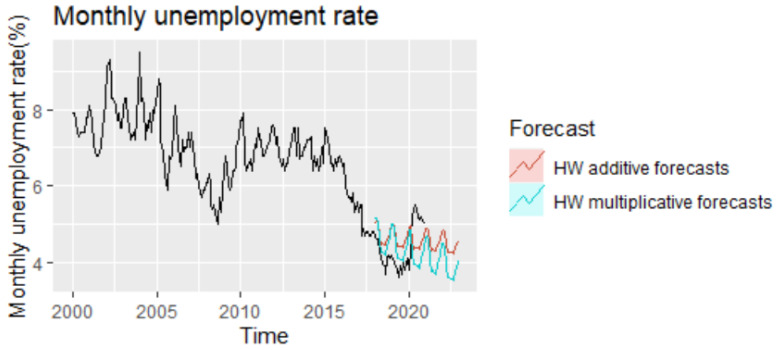
The forecast of unemployment rate based on Holt–Winters (HW) models for the period 2021–2022.

Analyzing the evolution of monthly unemployment rate for the period 2021–2022, it can be highlighted the fact that the forecast projections tend to under evaluate the actual series, not capturing the impact of the pandemics, and revealing a downward trend in both cases, which is more accentuated in the case of the multiplicative model (Figure 4).

### 4.2. ETS Models Results

In the process of obtaining a reliable forecast of the monthly unemployment rate, the ETS automatic selection framework, based on minimizing the AICc, revealed the optimal model to be an ETS(M,N,M) with multiplicative error, no trend, and multiplicative season. The empirical results highlighted that on the training dataset, the ETS model produces better results in comparison with HW additive or multiplicative methods (Table 3). The ETS(M,N,M) model will provide different point forecasts to the multiplicative Holt–Winters’ method, because the parameters have been estimated differently, the default estimation method being maximum likelihood rather than minimum sum of squares (Table 4).

**Figure 5:**
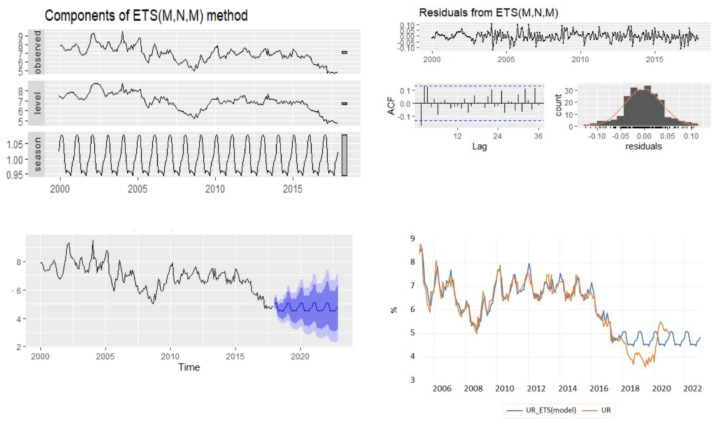
The forecast of unemployment rate based on the results of ETS(M,N,M).

The plot of ETS(M,N,M) components displays the states over time, while Figure 3 shows point forecasts and prediction intervals generated from the model. The empirical results of the model pointed out an under evaluation of the real values during the period of the test dataset from 2018 to 2020, highlighting an oscillating evolution characterized by a strong seasonal pattern also for the forecast projections period, 2021–2022 (Figure 5).

### 4.3. NNAR Model

**Figure 6:**
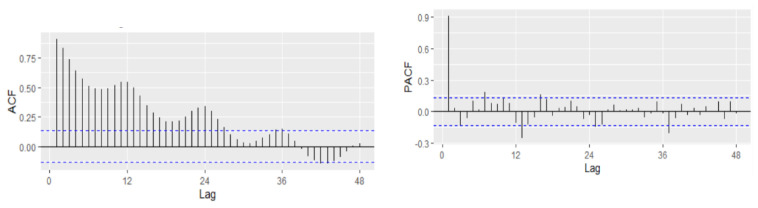
Autocorrelation and partial correlation plot of Romania’s monthly unemployment rate.

In order to fit the NNAR model, the series of unemployment rate has been explored on the training dataset in the process of identifying the order of an AR term present in the data, using the correlogram of the series. Based on the ACF and PACF plots, a pure AR(1) process can be highlighted for the non-seasonal part. Analyzing the ACF plot, the decaying spikes at every 12-month interval indicate a seasonal component present in the data (Figure 6). As the autocorrelation at the seasonal period (ACF at lag 12) is positive, an autoregressive model for the seasonal part should be considered; therefore, the order P was set to 1. Therefore, a NNAR(1,1,k)_12_ model is fitted, and the in-sample and out-sample root mean square error (RMSE), mean absolute error (MAE), mean absolute scale error (MASE), and mean absolute percentage error (MAPE) are provided in Table 5 where k = 1, …, 14.

**Figure 7:**
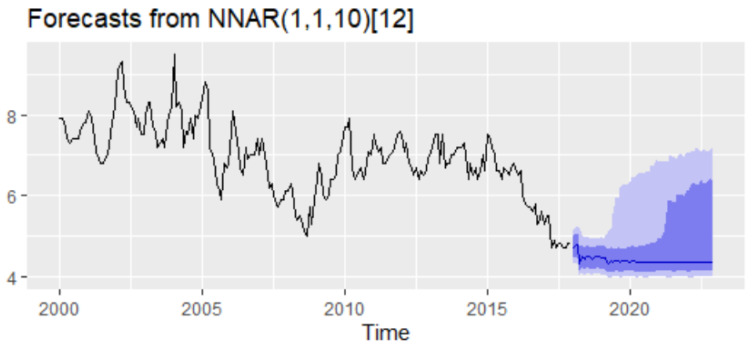
Forecasts from a neural network with one seasonal and non-seasonal lagged input and one hidden layer containing ten neurons.

The selection of the best model relied on the lowest values of all the forecast accuracy measures (RMSE, MAE, MAPE, and MASE), but especially on the values of MAPE and MASE, which are scale independent and used to compare forecast accuracy across series on different scales and seen as an appropriate measure when the out-of-sample data are not of the same length as the in-sample data. Based on the results of Table 5, MASE and MAPE are lower for the training dataset with 12 nodes in the hidden layer, whereas the out-of-sample MASE and MAPE are lower for 10 nodes in the hidden layer. Therefore, we can consider as the best choice the model NNAR(1,1,10)_12_. The forecast of the unemployment rate based on the NNAR(1,1,10)_12_ model results revealed a downward trend with a peak in September 2018 (4.43%) and with a forecasting value for 2021–2022 oscillating around the value of 4.35% (Figure 7).

### 4.4. SARIMA Model

**Figure 8:**
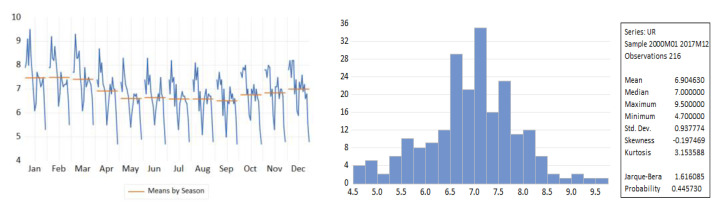
Descriptive statistics of unemployment rate for the horizon 2000–2017.

For fitting a SARIMA model, we used data covering the period January 2000 to December 2017. The descriptive statistics values of the unemployment rate for the training dataset are displayed in Figure 8. The series exhibited a strong seasonal pattern over the horizon 2000–2017.

#### 4.4.1. Testing for Non-Stationarity

**Figure 9:**
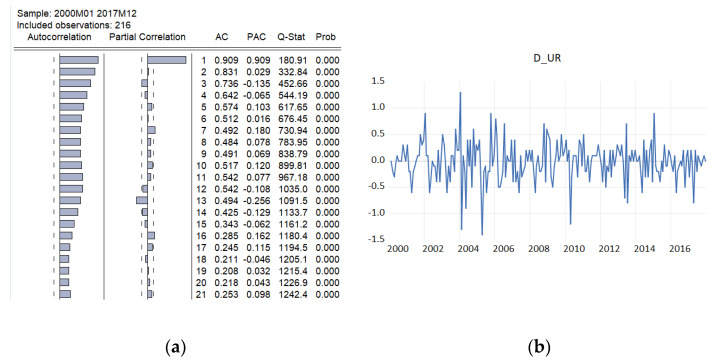
Autocorrelation and partial correlation plot of Romania’s monthly unemployment rate (**a**) and first difference of the original time series (**b**).

In order to fit a suitable time series model, the stationarity need to be investigated based on Augmented Dickey–Fuller and Philips–Perron tests. The graphical inspection of the autocorrelation and partial correlation plot of Romania’s quarterly unemployment rate (Figure 9) revealed that the values of autocorrelation coefficients decrease slowly, pointing out a nonstationary and relatively stable seasonal pattern of our time series.

The time-series plot of the first difference of the series highlighted that the unemployment rate is a non-stationary mean time series. The information is also confirmed by the empirical results of Bartlett and Ljung–Box tests.

The time-series plot of the first difference of the series highlighted that the first difference of the unemployment rate seems to be a stationary mean time series. Therefore, the original quarterly series is a non-stationary time series.

Diagram (b) from Figure 9 indicates that a possible stationarity exists in first differences. Alternately, we investigated the presence of unit roots by applying the Augmented Dickey–Fuller and Phillips–Peron tests initially to the series in level and then to the series in first differences. The empirical results on unemployment rate are displayed in Table 6, indicating that the series of unemployment rate is stationary in first differences, being integrated of order 1.

**Figure 10:**
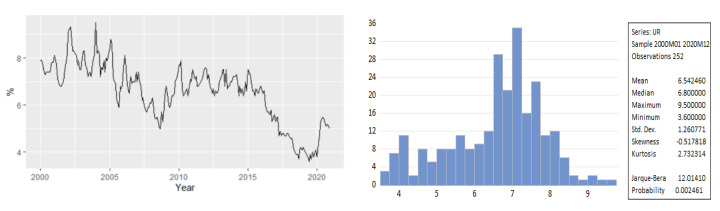
The Romanian ILO unemployment rate for the period 2000M1–2020M12.

The next step was to test the presence of a structural break around 2009 (from Figure 10), taking into account that the presence of a structural break will invalidate the results of unit root tests. Therefore, the Zivot–Andrews test has been used, the empirical result revealing that there is not enough evidence to reject both the null hypothesis that unemployment has a unit root with structural break in trend, and in both intercept and trend (Table 7).

Thus, the empirical results proved that the unemployment rate is non-stationary and integrated of order 1, I(1).

However, because the series of unemployment exhibits a seasonal pattern over the training period, the study will use a seasonal ARIMA model instead of non-seasonal models; therefore, it is necessary to check whether the seasonality is needed to be differenced or not, testing if the stochastic seasonality is present within the data, the empirical results of Hegy test revealing the rejection of seasonal unit root and the acceptance of only a non-seasonal unit root. Therefore, seasonal difference is not needed.

Therefore, we can conclude that the unemployment rate is a non-stationary series, without stochastic seasonality and integrated of order 1. Thus, the rate of unemployment will be modeled at the first difference of the series within the SARIMA model and self-exciting threshold autoregressive (SETAR) model.

#### 4.4.2. Identification of the Model

For the first difference of the UR, the model identification implies the identification of proper values of p, P, q, and Q using the ACF and PACF plot. The seasonal part of an AR or MA model will be seen in the seasonal lags. The ACF plot has a spike at lags 4 and 6 and an exponential decay starting from seasonal lag 12, suggesting a potential non-seasonal MA component-MA(4) or MA(6) (Table 8).

**Figure 11:**
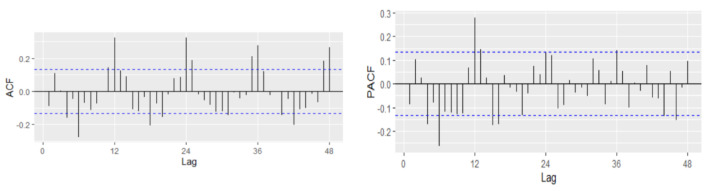
Autocorrelation and partial correlation plot of the first difference of the unemployment rate.

The PACF plot shows that lags 4, 6, and 12 are significant, capturing also potential non-seasonal AR components together with a seasonal AR(1) (Figure 11). In our case, because the autocorrelation at the seasonal lags (12, 24) is positive, a combination of seasonal and non-seasonal autoregressive models can be identified. Thus, several models have been specified, and based on AIC and BIC together with the goodness of fit measures, the best model has been identified.

Thus, several models have been specified, and based on AIC and BIC together with the goodness of fit measures, the best model has been identified, taking into account the lowest values of AIC and SBC. The best model has been an ARIMA(0,1,6)(1,0,1)_12_ considered based on the minimum value of AIC and SBC (Table 9).

#### 4.4.3. Model Estimation

Based on the model identified in the previous stage, we can proceed to the phase of model estimation using maximum likelihood method (ML), the empirical results being presented in Table 10. All coefficients statistically are significant at the 10% significance level.

#### 4.4.4. Diagnostic Checking of the Model

**Figure 12:**
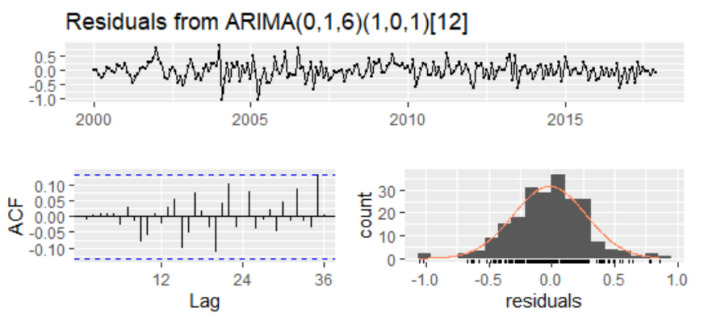
Diagnostic plot of SARIMA(0,1,6)(1,0,1)_12._

Apart from classical tests, the t-test for the statistical significance of the parameters, and the F-test for the validity of the model, the selection of the best model depends also on the performance of residuals. For that, the series of residuals has been investigated to follow a white noise. The empirical results of the Ljung–Box test show that the p-values of the test statistic exceed the 5% level of significance for all lag orders, which implies that there is no significant autocorrelation in residuals (Figure 12).

For checking autoregressive conditional heteroskedasticity (ARCH) in the residuals, the ARCH-LM test has been used, and the empirical results confirmed that there is no autoregressive conditional heteroscedasticity (ARCH) in the residuals (Table 11). Therefore, we can conclude that residuals are not autocorrelated and do not form ARCH models, the SARIMA(0,1,6)(1,0,1)_12_ model being reliable for forecasting (Table 12).

**Figure 13:**
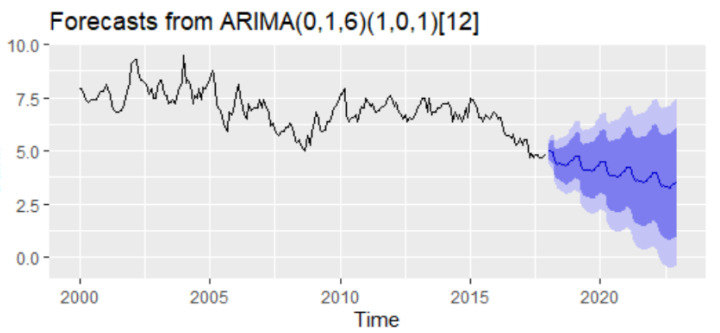
Forecasts of unemployment rate based on the results of ARIMA(0,1,6)(1,0,1)_12._

The forecast of the unemployment rate based on the ARIMA(0,1,6)(1,0,1)_12_ model results revealed a downward trend with a forecasting value for 2021–2022 oscillating around the value of 3–4% (Figure 13).

### 4.5. Self-Exciting Threshold Autoregressive (SETAR Model)

**Figure 14:**
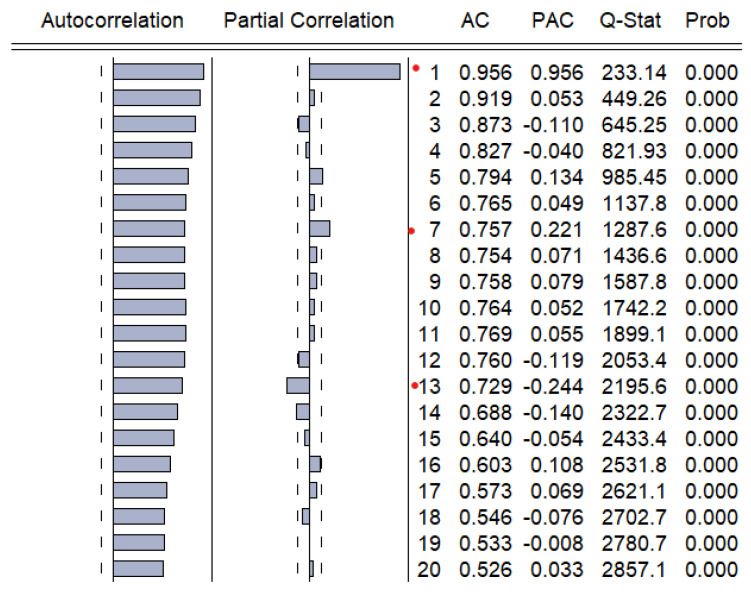
Partial autocorrelation plot of unemployment series.

In fitting a SETAR model for the Romanian unemployment rate, the first stages require the identification of the autoregressive order and testing the existence of nonlinear thresholds. The autoregressive order has been identified based on the PACF plot. Following Desaling [74], we explored the unemployment rate in level for identifying the lag autoregressive order, since the non-stationarity in UR does not cause the non-stationarity of nonlinear thresholds in the SETAR model, even if the existence of a unit root in one regime can occur. Significant spikes can be observed at lags 1, 7, and 13 (Figure 14).

At these lags, we have tested the presence of nonlinear thresholds applying the Tsay test of threshold nonlinearity, the empirical results being presented in Table 13, revealing that there is enough evidence to reject the null hypothesis of no nonlinear threshold in autoregressive order 1, 7, 8, 9, 10, 11, 12, and 13, the *p*-value being mostly less than 1%. Therefore, at these lags, the SETAR model is better than the simple AR model.

**Figure 15:**
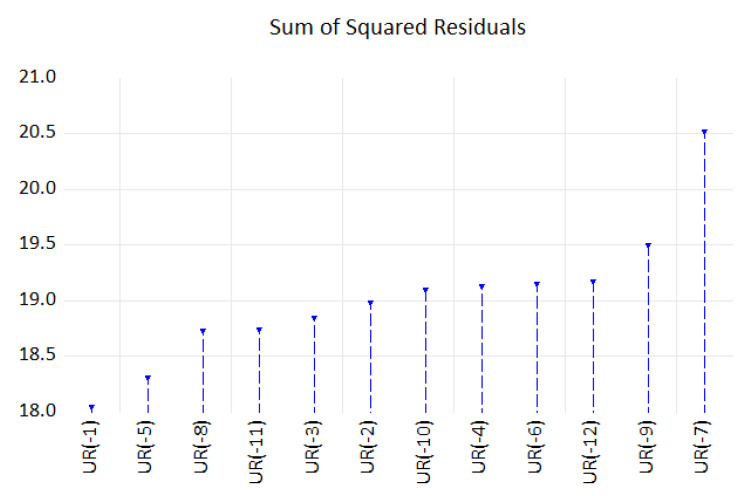
Grid search method estimation of one threshold value.

For the lags exhibiting a nonlinear threshold, we have used the lowest values of AIC to select the optimal model for which we will design the SETAR model. Thus, an AR(13) with possible values of delay parameter d = 1…12 < *p* has been used in setting the SETAR model. Since the number of potential regimes in the autoregressive model depends on the number of threshold values, a grid search method has been performed to determine the regimes and estimate the thresholds value under the condition of one threshold in AR based on the smallest value of sum of squared residuals. Thus, the delay parameter d = 10 registered the smallest value for residuals sum of squares; therefore, a SETAR model with two regimes of order 13 and threshold decay 1, a SETAR(2,13,1) model with a threshold variable could be appropriate to explain the nonlinearity in the data (Figure 15).

Table 14 displays the estimated parameters of the SETAR(2,13,1) with the threshold of 7.79, the model having the following specification:

$$y_{t}=\left\{\begin{array}{c} 0.13+0.82y_{t-1}+\ldots -0.307y_{t-13},\;if\;y_{t-1}<7.799\\ 2.344+0.539y_{t-1}+\ldots -0.019y_{t-13},\;if\;y_{t-1}>7.799 \end{array}\right\}$$.

After the estimation stage, the residuals of the model have been checked for best fit, verifying them for the information of serial autocorrelation, constant variance, and zero mean based on ARCH-LM and Breusch–Godgrey tests. Having the p-values greater than a 1% significance level, we can conclude that the residuals are not autocorrelated and with constant variance (Table 15).

**Figure 16:**
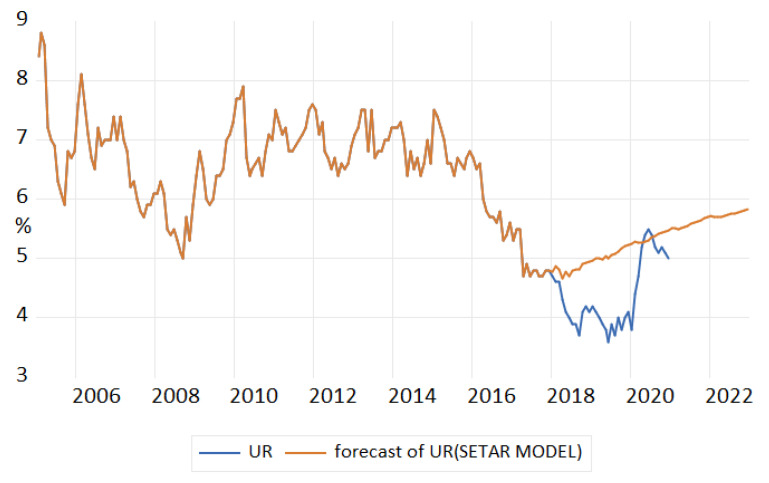
Forecasts of unemployment rate based on the results of the SETAR(2,13,1) model.

The forecast of unemployment rate based on the results of the SETAR(2,13,1) model (Table 16) revealed an upward trend, over evaluating the phenomenon (Figure 16).

### 4.6. Comparison of Models Forecasting Performance

Analyzing the forecasting performance of all models for the in-sample dataset based on RMSE, MAE, and MAPE as well as on the results of the Diebold and Marino test, it can observed that all three criteria suggested that multiplicative HW registered better forecast performance for the training dataset. The p-value of the Diebold and Marino test highlighted the existence of differences in forecast accuracy between almost all models, with the exception of multiplicative HW and ETS, for which the probability being higher than 10% does not provide enough evidence to reject the null hypothesis (Table 17).

The out-of-sample forecasting performance of models has performed with a one-step ahead recursive method. Based on RMSE and MAE values, the NNAR model has better forecasting performance, while MAPE stipulates the SARIMA model to register higher performance. For the out-of-sample data, the empirical results of the DM test pointed out differences in the predictive power for almost all models, with the exception of multiplicative HW and NNAR, for which the p-value is greater than the 10%, so the null hypothesis can not be rejected (Table 18).

**Figure 17:**
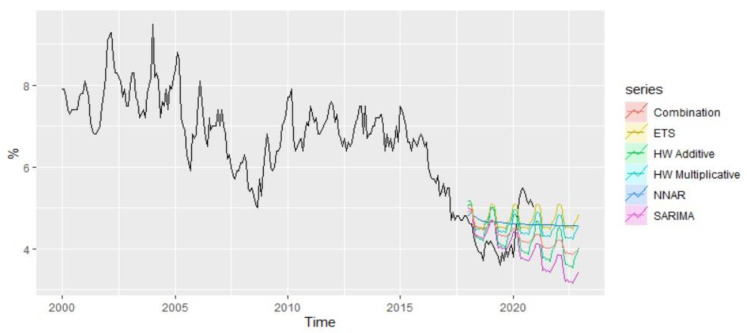
Forecast combination of the Romanian unemployment rate.

Analyzing comparatively the forecast performance of all methods during the period 2018–2022 and taking into account the presence of the pandemic shock, it is worth mentioning that ETS and Multiplicative HW are the methods that best capture the pandemic shock from 2020, offering forecast values relatively close to the real values of unemployment rate from the pandemics (Figure 17).

**Figure 18:**
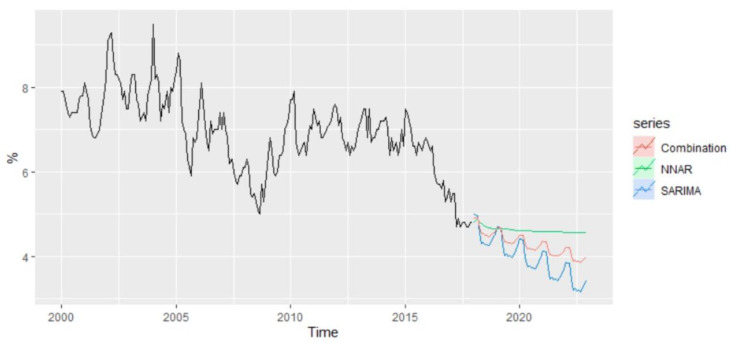
The forecasts of unemployment rate for the period 2021–2022.

Based on the methods offering the best results for out-of-sample forecasting, NNAR and SARIMA, the forecasted values of unemployment rate for the period 2021–2022 have been examined, revealing the existence of a slight difference (Figure 18).

According to NNAR, the predicted value of unemployment rate for January 2021 is estimated to be 4.35% compared with 5% in December 2020, and over the whole period, the forecast values oscillate around 4.35%. On the other hand, the forecast values based on the SARIMA model revealed a predicted value of 4.22% for the unemployment rate of January 2021 and highlighted a descending trend over the horizon 2021–2022, with a predicted value of 3.54% in December 2022.

An alternative to improving the forecast accuracy is to average the resulting forecasts based on these two methods, which are considered to be suitable for the modeling and forecasting of unemployment rate.

## 5. Conclusions

Making predictions about unemployment rate, one of the core indicators of the Romanian labor market with fundamental impact on the government future social policy strategies, is of great importance, mostly in this period of a major shock in the economy caused by the pandemic.

In this context, the aim of the research has been to evaluate the forecasting performance of several models and to build future values of unemployment rate for the period 2021–2022 using the most suitable results. In order to do that, we have employed exponential smoothing models, both additive and multiplicative Holt–Winters (HW) models together with an ETS model, the SARIMA model, the neural network autoregression (NNAR) model, and the SETAR model, which allow taking into account a nonlinear behavior and a switching regime on the time series and predicting future values of unemployment rate beyond the period under consideration.

The empirical results revealed for unemployment rate a non-stationary nonlinear and seasonal pattern in data. The out-of-sample forecasting accuracy of the models based on the performance measures RMSE and MAE pointed out the NNAR model as performing better, while MAPE indicated SARIMA to have the best performance. The empirical results of the Diebold–Mariano test at one forecast horizon for out-of-sample methods revealed differences in the forecasting performance between SARIMA and NNAR; of these, the best model of modeling and forecasting unemployment rate was considered to be the NNAR model.

## Figures and Tables

**Table 1:** The empirical results of HW for the forecast of unemployment rate.

Model 1: Holt–Winters’ Multiplicative Method	Model 2: Holt–Winters’ Additive Method
Smoothing parameters:	Smoothing parameters:
Alpha (level) = 0.6928	Alpha (level) = 0.7503
Beta (trend) = 0.0001	Beta (trend) = 0.0001
Gamma (seasonal) = 0.0001	Gamma (seasonal) = 0.0001
AIC = 630.187	AIC = 645.789
AICc = 633.278	AICc = 648.8807
BIC = 687.566	BIC = 703.169

**Table 2:** Forecasting performance of Holt–Winters.

	Holt–Winters’ Multiplicative Method		Holt–Winters’ Additive Method	
	Training Dataset	Testing Dataset	Training Dataset	Testing Dataset
ME	−0.0124	−0.2670	0.0006	−0.0371
RMSE	0.2771	0.6906	0.2804	0.7480
MAE	0.2086	0.6524	0.2109	0.6273
MPE	−0.3191	−7.8322	−0.1259	−2.6101
MAPE	3.0368	15.1393	3.0699	13.8268
MASE	0.3317	1.0374	0.3353	0.9974

**Table 3:** The empirical results of ETS (error, trend, seasonal) models for the forecast of unemployment rate.

ETS(M,N,M) Model: Multiplicative Error, No Trend, Multiplicative Season
Smoothing parameters:
Alpha(level) = 0.7914
Gamma(seasonal) = 0.0001
AIC = 627.799
AICc = 630.199
BIC = 678.428

**Table 4:** Forecasting performance of ETS model.

	ETS Model
	Training dataset
ME	−0.0166
RMSE	0.2788
MAE	0.2097
MPE	−0.3682
MAPE	3.0569
MASE	0.3335

**Table 5:** Forecasting performance of NNAR(1,1,k)_12_.

	Training Dataset				Test Dataset			
k	RMSE	MAE	MAPE	MASE	RMSE	MAE	MAPE	MASE
1	0.3570	0.2734	3.9654	0.4348	0.6792	0.6399	16.2143	1.0174
2	0.3477	0.2662	3.8562	0.4233	0.9019	0.8542	21.6274	1.3582
3	0.3402	0.2604	3.7626	0.4141	0.8510	0.8044	20.3754	1.2790
4	0.3329	0.2553	3.6772	0.4059	2.0452	1.8630	47.2547	2.9622
5	0.3297	0.2524	3.6264	0.4013	1.6242	1.4196	36.1478	2.2572
6	0.3228	0.2464	3.5341	0.3918	0.7710	0.7208	18.2993	1.1461
7	0.3195	0.2443	3.5057	0.3884	0.7739	0.7221	18.3387	1.1482
8	0.3173	0.2421	3.4737	0.3850	0.8042	0.7518	19.0849	1.1954
9	0.3167	0.2421	3.4681	0.3850	0.7873	0.7356	18.6744	1.1696
10	0.3150	0.2411	3.4513	0.3834	0.5979	0.5508	14.0168	0.8758
11	0.3087	0.2362	3.3860	0.3757	0.6936	0.6450	16.3913	1.0256
12	0.3033	0.2329	3.3456	0.3704	0.6220	0.5747	14.6184	0.9139
13	0.3058	0.2339	3.3533	0.3719	0.7008	0.6510	16.5462	1.0351
14	0.3064	0.2357	3.3779	0.3749	0.6944	0.6452	16.4001	1.0260

**Table 6:** Unit root analysis of the Romanian unemployment rate.

Variable	Unit Root [Transf.]		Level		First Difference	
			ADF	PP	ADF	PP
Unemployment rate	I(1) [∆UR]	T&C	−3.56 **	−3.52 **	−15.87 ***	−16.20 ***
		C	−2.58 *	−2.72 *	−15.90 ***	−16.01 ***
		None	−0.90	−0.98	−15.91 ***	−16.01 ***

Note: ***, **, and * means stationary at 1%, 5%, and 10%; T&C represents the most general model with a constant and trend; C is the model with a constant and without trend; None is the most restricted model without a drift and trend. For the ADF test, the number of lags was determined using SCH criterion for a maximum of 14 lags to remove serial correlation in the residuals. For both PP tests, the value of the test was computed using Newey–West Bandwith (as determined by Bartlett kernel). Tests for unit roots have been carried out in E-VIEWS 11.

**Table 7:** Zivot–Andrews unit root test having a structural break for unemployment rate.

Series (Trend Specification: Trend and Intercept)		Allowing for Break in Trend	Allowing for Break in Both Intercept and Trend
Unemployment Rate	Minimum t-stat (Lag length has been established using SBC criterion for maximum 14 lags) *p*-value	−4.139 (0.13)	−4.484 (0.243)
	Critical values		
	1%	−5.067	−5.719
	5%	−4.524	−5.175
	10%	−4.261	−4.893
		Potential break point at 2015M09	Potential break point at 2009M06

**Table 8:** HEGY test of seasonality for level of unemployment series.

Null	Simulated *p*-Value *	The Presence of Non-Seasonal Unit Root **	The Presence of Seasonal Unit Root **
Unemployment rate Non-seasonal unit root (zero frequency) Seasonal unit root (2 months per cycle) Seasonal unit root (4 months per cycle)	0.736310 0.005643 0.000000	Yes	No
Seasonal unit root (2.4 months per cycle)	0.000177		
Seasonal unit root (12 months per cycle)	0.000177		
Seasonal unit root (3 months per cycle)	0.000000		
Seasonal unit root (6 months per cycle)	0.000000		

Note: The HEGY test was applied taking into account intercept and trend and seasonal dummies; the maximal number of lags was eight following Schwarz criterion and a number of 1000 simulations. * If the probability is higher than 0.10, then the presence of the non-seasonal unit root cannot be rejected. ** If the probability is higher than 0.10, then the presence of a seasonal unit root cannot be rejected.

**Table 9:** AIC and SBC for the suggested ARIMA models.

Model	AIC	AICc	BIC
ARIMA(4,1,4)(1,0,0)_12_	133.2	134.28	166.9
ARIMA(4,1,4)(2,0,0)_12_	129.99	131.29	167.07
ARIMA(4,1,4)(3,0,0)_12_	124.03	125.58	164.48
ARIMA(4,1,4)(3,0,1)_12_	116.73	118.54	160.55
ARIMA(0,1,4)(3,0,0)_12_	148.39	149.09	175.36
ARIMA(4,1,4)(0,0,3)_12_	130.87	132.41	171.31
ARIMA(4,1,4)(0,0,1)_12_	136.36	137.43	170.06
ARIMA(6,1,0)(1,0,0)_12_	148.51	149.21	175.48
ARIMA(6,1,0)(2,0,0)_12_	132.34	133.22	162.68
ARIMA(6,1,0)(3,0,0)_12_	124.33	125.41	158.04
ARIMA(6,1,6)(3,0,0)_12_	128.28	131.03	182.21
ARIMA(0,1,6)(1,0,0)_12_	146.22	146.92	173.19
ARIMA(0,1,6)(2,0,0)_12_	131.15	132.03	161.49
ARIMA(0,1,6)(3,0,0)_12_	124.17	125.25	157.87
ARIMA(0,1,6)(1,0,1)_12_	108.42	109.3	138.76
ARIMA(0,1,6)(2,0,1)_12_	109.83	110.91	143.54

**Table 10:** Estimates of parameters for SARIMA(0,1,6)(1,0,1)_12._

	Estimate S	td. Error	z Value	Pr(>|z|)
ma6	−0.12316	0.069532	−1.7712	0.07653 *
sar1	0.983605	0.015399	63.8766	2.2 × 10^–16^ ***
sma1	−0.8462	0.066935	−12.6421	2.2 × 10^–16^ ***

Note: *** and * means stationary at 1% and 10%.

**Table 11:** Empirical results of JB test and autoregressive conditional heteroskedasticity (ARCH)-LM test for model residuals.

	Ljung–Box Test	*p*-Value	ARCH-LM Test	*p*-Value
12	2.9459	0.5669	9.1184	0.6928
24	15.123	0.5157	44.267	0.2345
36	25.531	0.5988	51.336	0.1878
48	40.434	0.4511	58.159	0.1495

**Table 12:** Forecasting performance of SARIMA(0,1,6)(1,0,1)_12._

	Training Dataset	Testing Dataset
RMSE	0.28861	0.764092
MAE	0.22163	0.615342
MAPE	3.20478	13.37031
MASE	0.35240	0.97840

**Table 13:** The empirical results of the Tsay test.

Order	F-Statistics	*p*-Value	AIC
AR(1)	4.798	0.029 **	0.734
AR(2)	1.935	0.125	-
AR(3)	1.363	0.231	-
AR(4)	1.097	0.366	-
AR(5)	1.119	0.341	-
AR(6)	1.267	0.202	-
AR(7)	1.744	0.016 **	0.689
AR(8)	1.994	0.001 ***	0.693
AR(9)	2.116	0.001 ***	0.697
AR(10)	1.989	0.001 ***	0.696
AR(11)	2.151	0.001 ***	0.698
AR(12)	2.257	0.001 ***	0.702
AR(13)	2.034	0.003 ***	0.628

Note: ***, ** means statistical significance at 1%, 5%.

**Table 14:** Estimates of parameters for SETAR(2,13,1).

Variable	Coefficient	Std. Error	Prob.
UR(−1) < 7.7999999–171 obs			
C	0.130	0.264	0.623
UR(−1)	0.820	0.079	0.000 ***
UR(−2)	0.254	0.099	0.011 ***
UR(−3)	−0.055	0.099	0.579
UR(−4)	−0.136	0.098	0.168
UR(−5)	0.063	0.092	0.499
UR(−6)	−0.126	0.092	0.172
UR(−7)	0.078	0.092	0.401
UR(−8)	0.069	0.097	0.475
UR(−9)	0.010	0.093	0.917
UR(−10)	−0.030	0.099	0.762
UR(−11)	0.103	0.101	0.310
UR(−12)	0.237	0.099	0.018 **
UR(−13)	−0.307	0.076	0.000 ***
7.7999999 <= UR(−1)–32 obs			
C	2.344	2.092	0.264
UR(−1)	0.539	0.202	0.008 ***
UR(−2)	0.214	0.184	0.247
UR(−3)	0.022	0.194	0.909
UR(−4)	−0.643	0.207	0.002 ***
UR(−5)	0.682	0.335	0.043
UR(−6)	0.062	0.264	0.814
UR(−7)	0.141	0.299	0.637
UR(−8)	−0.639	0.267	0.018 **
UR(−9)	−0.526	0.301	0.083
UR(−10)	0.479	0.218	0.030 **
UR(−11)	0.833	0.181	0.000 ***
UR(−12)	−0.440	0.257	0.089
UR(−13)	−0.019	0.198	0.923

Note: ***, ** means statistical significance at 1%, 5%.

**Table 15:** Residuals diagnostic test for SETAR(2,13,1).

	BG Test (F-Stat)	*p*-Value	ARCH-LM Test	*p*-Value
12	1.180	0.301	0.722	0.728
24	0.99	0.473	0.738	0.805
36	1.179	0.247	0.991	0.493
48	1.197	0.213	1.068	0.381

**Table 16:** Forecasting performance of SETAR(2,13,1).

	Training Data Set	Testing Data Set
RMSE	0.931	0.834
MAE	0.803	0.715
MAPE	11.598	17.742
MASE	12.022	15.770

**Table 17:** In-sample forecasting performance of models.

Measures	Model					
	Holt–Winters Additive	Holt–Winters Multiplicative	ETS	NNAR	SARIMA	SETAR
RMSE	0.2804	0.2771	0.2788	0.315	0.28861	0.931
MAE	0.2109	0.2086	0.2097	0.2411	0.22163	0.803
MAPE	3.0699	3.0368	3.0569	3.4513	3.20478	11.598
**DM Test for in Sample at h = 1**						
**Models**		**DM Test Statistics**		***p*** **-Value**		
HW Additive vs. HW Multiplicative		7.7819		0		
HW Additive vs. ETS		−7.7841		0		
HW Additive vs. NNAR		4.2089		0		
HW Additive vs. SARIMA		1.6588		0.0986		
HW Additive vs. SETAR		55.592		0		
HW Multiplicative vs. ETS		0.3324		0.7399		
HW Multiplicative vs. NNAR		8.1815		0		
HW Multiplicative vs. SARIMA		8.0321		0		
HW Multiplicative vs. SETAR		55.568		0		
ETS vs. NNAR		8.1791		0		
ETS vs. SARIMA		8.0342		0		
ETS vs. SETAR		55.568		0		
NNAR vs. SARIMA		−3.3088		0.001		
NNAR vs. SETAR		54.421		0		
SARIMA vs. SETAR		−55.615		0		

**Table 18:** Out-of-sample forecasting performance of models.

Measures	Model					
	Holt–Winters Additive	Holt–Winters Multiplicative	ETS	NNAR	SARIMA	SETAR
RMSE	0.748	0.6906		0.5979	0.764092	0.834
MAE	0.6273	0.6524		0.5508	0.615342	0.715
MAPE	13.8268	15.1393		14.0168	13.37031	17.742
**DM Test for Out of Sample at h = 1**						
**Models**		**DM Test Statistics**		***p*** **-Value**		
HW Additive vs. HW Multiplicative		−13.541		0		
HW Additive vs. ETS		14.388		0		
HW Additive vs. NNAR		7.4791		0		
HW Additive vs. SARIMA		16.703		0		
HW Additive vs. SETAR		−11.61		0		
HW Multiplicative vs. ETS		13.616		0		
HW Multiplicative vs. NNAR		1.4745		0.1457		
HW Multiplicative vs. SARIMA		17.175		0		
HW Multiplicative vs. SETAR		−16.362		0		
ETS vs. NNAR		−3.2896		0.0016		
ETS vs. SARIMA		−15.773		0		
ETS vs. SETAR		17.254		0		
NNAR vs. SARIMA		−12.841		0		
NNAR vs. SETAR		18.072		0		
SARIMA vs. SETAR		−17.303		0		

## Data Availability

Publicly available datasets were analyzed in this study. This data can be found here: https://ec.europa.eu/eurostat/en/web/products-datasets/-/UNE_RT_M (accessed on 15 January 2021).
